# Potential profile analysis of return-to-work readiness and self-efficacy in postoperative breast cancer patients who have returned to work

**DOI:** 10.3389/fonc.2026.1755456

**Published:** 2026-07-03

**Authors:** Qiu Huo, Shiqi Li, Yunxia Pu, Ying Huang, Huizhuo Deng

**Affiliations:** 1 West China School of Public Health and West China Fourth Hospital, Sichuan University, Chengdu, China; 2 Department of Biotherapy Research, West China Hospital, Sichuan University, Chengdu, Sichuan, China; 3 Department of Oncology, West China Hospital JinCheng, Sichuan University, Chengdu, Sichuan, China

**Keywords:** influencing factors, latent profile analysis, postoperative breast cancer, return-to-work readiness, return-to-work self-efficacy

## Abstract

**Objective:**

To identify the latent profiles of return-to-work readiness (RRTW) and return-to-work self-efficacy (RTW-SE) among postoperative breast cancer patients who have returned to work, and to analyze their influencing factors, thereby providing a basis for improving their work-related quality of life.

**Methods:**

A cross-sectional study was conducted using convenience sampling to recruit 173 breast cancer patients from a tertiary Grade A hospital in Chengdu, China. All participants had returned to work for at least 3 months post-surgery. Data were collected using a general information questionnaire, the 9-item Franche RRTW Scale, and the 11-item Lagerveld RTW-SE Scale. Latent profile analysis (LPA) was employed to identify distinct subgroups, and logistic regression analysis was used to examine the influencing factors.

**Results:**

LPA identified two distinct latent profiles: The “Uncertain Maintenance-Low Self-Efficacy” group (34.68%): Characterized by high scores on the Uncertain Maintenance dimension of RRTW and low levels of RTW-SE. The “Proactive Maintenance-High Self-Efficacy” group (65.32%): Characterized by high scores on the Proactive Maintenance dimension of RRTW and high levels of RTW-SE. Multivariate logistic regression analysis revealed that a daily working time of 6–8 hours (odds ratio [OR] = 2.303, 95% confidence interval [CI]: 1.058-5.015), older age (OR = 1.062, 95% CI: 1.003-1.125), and higher work satisfaction (OR = 2.078, 95% CI: 1.319-3.271) were significantly associated with belonging to the high self-efficacy profile (all P < 0.05).

**Conclusion:**

Postoperative breast cancer patients who have returned to work exhibit significant heterogeneity in their RRTW and RTW-SE profiles. Targeted intervention strategies are necessary for younger patients, those with longer working hours, and those with lower job satisfaction. Optimizing work schedules, enhancing job satisfaction, and providing enhanced psychological support for younger patients are recommended to facilitate better work adaptation.

## 1 Introduction

Breast cancer (BC) is the most common malignant tumor among women. In recent years, there has been a trend of rising incidence rates in younger populations. Reports indicate that the high-risk age group for adult female breast cancer patients in China is 45–55 years (1), and approximately 70% of diagnosed breast cancer patients are adults who are still of working age (2). For cancer patients within the working-age population, occupational and work-related issues play a crucial role in their lives (3). Studies have confirmed that return-to-work (RTW) for breast cancer patients is associated with lower all-cause mortality (4), indicating a better rehabilitative status. However, breast cancer patients in China overall face challenges in occupational reintegration: on one hand, the RTW rate is relatively low; on the other hand, even among those who have successfully returned to work, their work-related quality of life remains a concern (5), a study by Chen Sinuo et al. (6) showed that the work-related quality of life of postoperative breast cancer patients in China after RTW needs improvement and is lower than that in other countries, with self-efficacy positively correlating with quality of life. A qualitative study by Petersen et al. (7) revealed that most cancer survivors are affected by various factors preventing them from sustaining work. Research by Wang Liying (8) also indicated that most patients are either unable to maintain normal work or develop a strong sense of uncertainty towards high-intensity work. Therefore, identifying and thoroughly investigating the key predictive and protective factors influencing the maintenance of a good work status and work-related quality of life in patients who have returned to work is particularly important. Currently, however, most research focuses on identifying reasons for the low RTW rate among Chinese breast cancer patients and strategies for improvement, with less attention given to those who have already returned to work.

Return-to-work readiness (RRTW) refers to a patient’s preparedness to return to their original job or take on new work following the onset of an illness (9). Studies have found that a patient’s level of RRTW can effectively predict their work participation after rehabilitation (10) and their ability to maintain employment after returning to work (9). Return-to-work self-efficacy (RTW-SE), a crucial predictor of successful return to work, is defined as a patient’s confidence in their ability to resume work and perform job-related tasks (11). Research has demonstrated that RTW-SE is positively correlated with work ability and employment status (12).

RRTW and RTW-SE are conceptually closely related concepts, both focusing on an individual’s psychological preparedness for a successful return to the workplace (13). Specifically, RTW-SE is widely regarded as a psychological and cognitive factor that both predicts and constitutes RRTW (14). However, there are important distinctions in their conceptual focus and scope. Compared to RTW-SE, RRTW represents a broader and more comprehensive construct. It not only encompasses confidence in one’s own capabilities (i.e., RTW-SE), but may also include dimensions such as motivation levels for returning to work, perceived workplace support, expectations regarding post-return outcomes, and the temporal planning of the actual return behavior (15). Therefore, RTW-SE and RRTW are related yet distinctly different.

For patients who have already returned to work, readiness for return-to-work primarily refers to the maintenance phase after their return, which includes the uncertain maintenance stage and the proactive maintenance stage. The uncertain maintenance stage refers to a condition in which, although patients have returned to work, their work status remains relatively poor, with a risk of subsequent work withdrawal. In contrast, the proactive maintenance stage refers to a condition in which patients have returned to work and demonstrate high work engagement, enabling them to maintain a stable work status (16). Return-to-work self-efficacy, as a critical psychosocial core construct, serves as a predictor of return-to-work status and is significantly associated with the degree of RTW maintenance (17). A higher proportion of patients with higher self-efficacy scores are in the proactive maintenance stage (18). A longitudinal cohort study of workers returning to work conducted by Black et al. (19) demonstrated that return-to-work self-efficacy significantly predicted the maintenance status of return-to-work at four to six months. Furthermore, a study by Hu Yaoyao et al. (20) also reported that return-to-work self-efficacy can be used to predict patients’ maintenance status after returning to work. Therefore, we hypothesize that the proactive maintenance stage is positively associated with self-efficacy, whereas the uncertain maintenance stage is negatively associated with self-efficacy.

Currently, studies investigating the factors influencing RRTW and RTW-SE in breast cancer patients predominantly employ a “variable-centered” analytical approach (e.g., using total or subscale scores), which often overlooks the reality of how individual heterogeneity dynamically influences RTW behavior (21). To address this gap, this study aims to utilize latent profile analysis (LPA) to identify distinct latent profiles among breast cancer patients who have returned to work, based on their characteristics in RRTW and RTW-SE, and to analyze the influencing factors for these different profiles. The findings are intended to inform the development of targeted intervention strategies to improve their work-related quality of life. LPA is a person-centered method that classifies individuals into latent subgroups (profiles) based on their response patterns to manifest variables, such as RRTW and RTW-SE (22). This approach helps uncover heterogeneity within the population of breast cancer patients who have returned to work by identifying subgroups with distinct patterns of RRTW and RTW-SE. Consequently, it establishes a foundation for further analyzing differences between profiles in maintaining work status and work-related quality of life, as well as their unique influencing factors, thereby providing a theoretical basis for precise interventions.

## 2 Subjects and methods

### 2.1 Study participants

This study employed a cross-sectional design. Participants were recruited via convenience sampling from breast cancer patients who had undergone surgery at a tertiary Grade A hospital in Chengdu between March and October 2024.

Inclusion criteria were as follows: (1) aged 18–60 years; (2) diagnosed with breast cancer (Stage I, II, or III) confirmed by histopathology; (3) under follow-up and had returned to work for at least 3 months; (4) mentally clear and able to understand and complete the questionnaire accurately; (5) provided informed consent and voluntarily participated in the study.
Exclusion criteria included: (1) presence of other malignant tumors; (2) comorbid psychiatric disorders, hearing impairment, or severe physical disabilities; (3) receiving palliative care.

The sample size for this Latent Profile Analysis (LPA) was estimated to require 100–150 participants, based on recommendations from previous studies (23). Additionally, according to the standard calculation method for sample size in studies analyzing influencing factors (24), the required sample size should be 5–10 times the number of independent variables. With 18 independent variables in this study and an anticipated 20% invalid questionnaire rate, the target sample size ranged from 110 to 220. A total of 180 questionnaires were distributed. After excluding 7 invalid responses, 173 valid questionnaires were retained, yielding a valid response rate of 96.1%. This study was approved by the Hospital Ethics Committee (Approval No: [2023] Ethics (1834)).

### 2.2 Methods

#### 2.2.1 Survey instruments

##### 2.2.1.1 General information questionnaire

A General Information Questionnaire was developed by the researchers based on a literature review and expert opinions. It encompassed the following domains: Demographic data: e.g., age, educational level, marital status, economic status, personality; Work-related information: e.g., occupation, type of employer, job attributes, working hours, job intensity, job satisfaction; Disease-related information: e.g., TNM (Tumor Node Metastasis) pathological stage, treatment modalities, surgical approach, radiotherapy, and chemotherapy.

##### 2.2.1.2 Return-to-work readiness scale

The Return-to-Work Readiness Scale was developed by Franche et al. (25). The original scale consists of two parts; this study utilized the second part, designed to assess patients who have already returned to work. This section contains 9 items across 2 dimensions: Proactive Maintenance (items 14, 15, 16, 17) and Uncertain Maintenance (items 18, 19, 20, 21, 22; note that item 21 is reverse-scored). Responses are recorded on a 5-point Likert scale, ranging from 1 (“strongly disagree”) to 5 (“strongly agree”). The evaluation principle is as follows: after summing the scores for items within each dimension, the dimension with the highest total score indicates the patient’s current stage. A higher stage represents a greater level of readiness. The Chinese version of the scale demonstrated good reliability, with Cronbach’s α coefficients ranging from 0.75 to 0.84 and test-retest reliability ranging from 0.80 to 0.83 for its dimensions.

##### 2.2.1.3 Return-to-work self-efficacy scale

The Return-to-Work Self-Efficacy (RTW-SE) Scale was developed by Dutch scholars Lagerveld et al. (26). This unidimensional scale comprises 11 items. It includes eight positively phrased items (1, 3, 4, 5, 7, 8, 10, 11) and three reverse-scored items (2, 6, 9). Responses are measured on a 6-point Likert scale, ranging from 1 (“strongly disagree”) to 6 (“strongly agree”). For scoring, items 2, 6, and 9 are first reverse-scored. Subsequently, the scores of all 11 items are summed and divided by 11 to obtain a mean score, which represents the final scale score. A higher score indicates a greater level of RTW-SE and a greater likelihood of returning to work within three months. The Chinese version of the RTW-SE demonstrated good psychometric properties, with a content validity index of 0.91, a Cronbach’s α coefficient of 0.923, and a test-retest reliability of 0.799.

#### 2.2.2 Data collection and quality control

Data were collected using questionnaires administered by two masters of nursing students who underwent standardized training. Prior to the survey, patients were informed of its purpose, significance, methodology, and the principle of confidentiality. They received instructions on how to complete the questionnaire and key points to note. Questionnaires were filled out truthfully by the patients after they fully understood the requirements. For patients unable to complete the forms independently, assistance was provided by the investigators. A standardized script was used throughout the process to address any patient inquiries. All questionnaires were distributed and collected on-site.

#### 2.2.3 Statistical analysis

##### 2.2.3.1 Latent profile analysis

Latent profile analysis (LPA) was performed using Mplus 8.3 (Muthén & Muthén, 1998–2019) to identify distinct subgroups of postoperative breast cancer patients who had returned to work, based on their responses to the 9−item Return−to−Work Readiness (RRTW) scale and the 11−item Return−to−Work Self−Efficacy (RTW−SE) scale. All models were estimated using the raw data under the missing at random (MAR) assumption, with missing values (coded 99) handled by full information maximum likelihood (FIML) Model fit was evaluated using the following three types of indicators:

Model estimation: The robust maximum likelihood estimator (MLR) was used to account for potential non−normality of the indicators. To avoid local maxima, each model was run with 200 initial−stage random starts and 50 final−stage optimizations, retaining the solution with the highest log−likelihood. The Expectation-Maximization (EM) algorithm convergence criteria were set to a log−likelihood change tolerance of 1e−6 and a relative log−likelihood change tolerance of 1e−6.

Scaling of indicators: All items were entered into the LPA using their raw scores without standardization, because the two scales have comparable ranges (5−point vs. 6−point Likert). The model assumed equal variances across latent classes (homogeneity restriction), which is the default setting in Mplus for continuous indicators.

Model selection: A series of models with 1 to 4 profiles was estimated. The optimal number of profiles was determined using the following criteria:

1. Information criteria: Akaike information criterion (AIC), Bayesian information criterion (BIC), and sample−size adjusted BIC (aBIC); lower values indicate better fit.
2. Classification quality: Entropy (values closer to 1 indicate clearer class separation) and average posterior class probabilities (recommended > 0.80).
3. Likelihood−ratio tests: Lo−Mendell−Rubin adjusted likelihood ratio test (LMR−LRT) and bootstrap likelihood ratio test (BLRT). A significant p−value (p < 0.05) for a k−profile model compared to a (k−1) profile model supports the k−profile solution.
4. Practical utility: each profile should contain at least 5% of the sample and be theoretically interpretable.

After selecting the optimal model, participants were assigned to their most likely latent class based on posterior probabilities. The entropy value and classification probabilities are reported to evaluate the accuracy of class assignment.

##### 2.2.3.1 Other statistical analyses

All other statistical analyses were performed using SPSS 26.0. Continuous data are presented as mean ± standard deviation. Group comparisons for normally distributed data were conducted using the t-test, while the Mann-Whitney U test was used for non-normally distributed data. Categorical data are expressed as numbers and percentages, and group comparisons were made using the Chi-square test. Logistic regression analysis was performed with the LPA-derived profile membership as the dependent variable and patients’ general characteristics as independent variables. The statistical significance level was set at α = 0.05.

## 3 Results

### 3.1 General characteristics of the participants

A total of 173 postoperative breast cancer patients were included in this study. The age of the participants ranged from 23 to 58 years, with a mean (standard deviation, SD) age of 41.14 (6.16) years. Detailed demographic and clinical characteristics are presented in **Table 1**.

**Table 1:** General characteristics of postoperative patients with breast cancer who have returned to work (N=173).

Item		Mean ± SD	Number(n)	Percentage (%)
Age (years)		41.14 ± 6.16		
Education	*- Elementary school or below*		2	1.16
	*- Junior high school*		13	7.51
	*- High school/vocational*		11	6.36
	*- College*		38	21.97
	*- Bachelor’s degree*		94	54.34
	*-Master’s degree or above*		15	8.67
Ethnicity	*-Han*		167	96.53
	*-Tibetan*		1	0.58
	*-Yi*		1	0.58
	*-Hui*		1	0.58
	*-others*		3	1.73
Marital Status	*- Single*		11	6.36
	*- Married*		149	86.13
	*- Divorced*		13	7.51
Per Capita Household Income	*≤3000*		6	3.47
	*3000-5000*		26	15.03
	*5000-8000*		35	20.23
	*8000-10000*		27	15.61
	*≥10000*		79	45.66
Personality	*-Typical Introvert*		8	4.62
	*-Introvert Leaning*		33	19.08
	*-Intermediate*		83	47.98
	*-Extrovert Leaning*		40	23.12
	*-Typical Extrovert*		9	5.20
Work Type	*- Physical labor*		6	3.47
	*- Mental labor*		110	63.58
	*- Mixed labor*		57	32.95
Employment Contract	*- Permanent staff*		45	26.01
	*- Contract-based staff*		99	57.23
	*- Other employment types*		29	16.76
Years of employment, yr		17.24 ± 8.12		
Professional Title	*- None*		84	48.55
	*- Junior Title*		18	10.40
	*- Intermediate Title*		46	26.59
	*- Senior Title*		20	11.56
	*- Other*		5	2.89
Employment status	*Partial return to work*		89	51.44
	*Full return to work*		73	42.20
	*New job*		11	6.36
Work Hours	*- <6 h/day*		18	10.40
	*- 6–8 h/day*		97	56.07
	*- >8 h/day*		58	33.53
Workload	*- Low*		10	5.78
	*- Moderate*		109	63.01
	*- High*		54	31.21
Job Satisfaction	*- Very dissatisfied*		6	3.47
	*- Dissatisfied*		12	6.94
	*- Neutral*		56	32.37
	*- Satisfied*		90	52.02
	*- Very satisfied*		9	5.20
Disease Duration	*- <1 year*		92	53.18
	*- 1–3 years*		68	39.31
	*- >3 years*		13	7.51
TNM Stage	*- Stage I*		47	27.33
	*- Stage II*		86	50.00
	*- Stage III*		21	12.21
	*- Unknown*		18	10.47
Surgery	*Yes*		173	100.00
Radiotherapy	*No*		47	27.17
	*Yes*		126	72.83
Chemotherapy	*No*		26	15.03
	*Yes*		147	84.97
Endocrine therapy	*No*		55	31.79
	*Yes*		118	68.21
Immunotherapy/Targeted therapy	*No*		125	72.25
	*Yes*		48	27.75
Surgery Type	*- Breast-conserving*		64	36.99
	*- Mastectomy*		44	25.43
	*- Reconstruction*		65	37.57
Lymph Dissection	*- None*		12	6.94
	*- Sentinel*		91	52.60
	*- Dissection*		70	40.46

### 3.2 The models were comprehensively compared based on model fit evaluation criteria and clinical interpretability

Latent profile analysis was performed using the scores from the 9-item Chinese version of the RRTW scale (for patients who have returned to work) and the 11-item RTW-SE scale. Models with 1 to 4 profiles were estimated sequentially, starting from a baseline 1-profile model. The fit indices for these competing models are presented in **Table 2**. Prior to conducting the latent profile analysis (LPA), we examined potential multicollinearity between the RRTW and RTW-SE items. All items from both scales were simultaneously entered into a linear regression model. Collinearity diagnostics indicated that variance inflation factors (VIFs) for all items ranged from 1.309 to 4.128, all below the conventional threshold of 5. These results confirm that no severe multicollinearity exists between the two constructs, justifying their simultaneous inclusion in the LPA.

**Table 2:** Model fit indices for latent profile analysis of RRTW and RTW-SE (N = 173).

Number of classes	AIC	BIC	aBIC	Entropy	LMR	BLRT	Class probabilities
1	9323.419	9449.551	9322.889				1
2	8388.731	8581.082	8387.922	0.952	0.001*	0.000*	0.34682/0.65318
3	7918.455	8177.025	7917.367	0.970	0.392	0.000*	0.26012/0.13295/0.60694
4	7770.210	8094.999	7768.844	0.949	0.701	0.000*	0.20231/0.23121/0.43353/0.13295

An asterisk (*) indicates statistical significance at p < 0.05.*.

Comparison of latent class models based on statistical and clinical criteria.

The results indicated that: (1) As the number of profiles increased, the AIC, BIC, and aBIC values decreased progressively, and the Entropy values were all > 0.8; (2) Using the criterion of a significant LMR-LRT test (*p* < 0.05), the 2-profile model was deemed optimal. Consequently, the 2-profile model was selected to represent the latent profiles of RRTW and RTW-SE among postoperative breast cancer patients who had returned to work.

### 3.3 Naming the latent profiles

**Figure 1:**
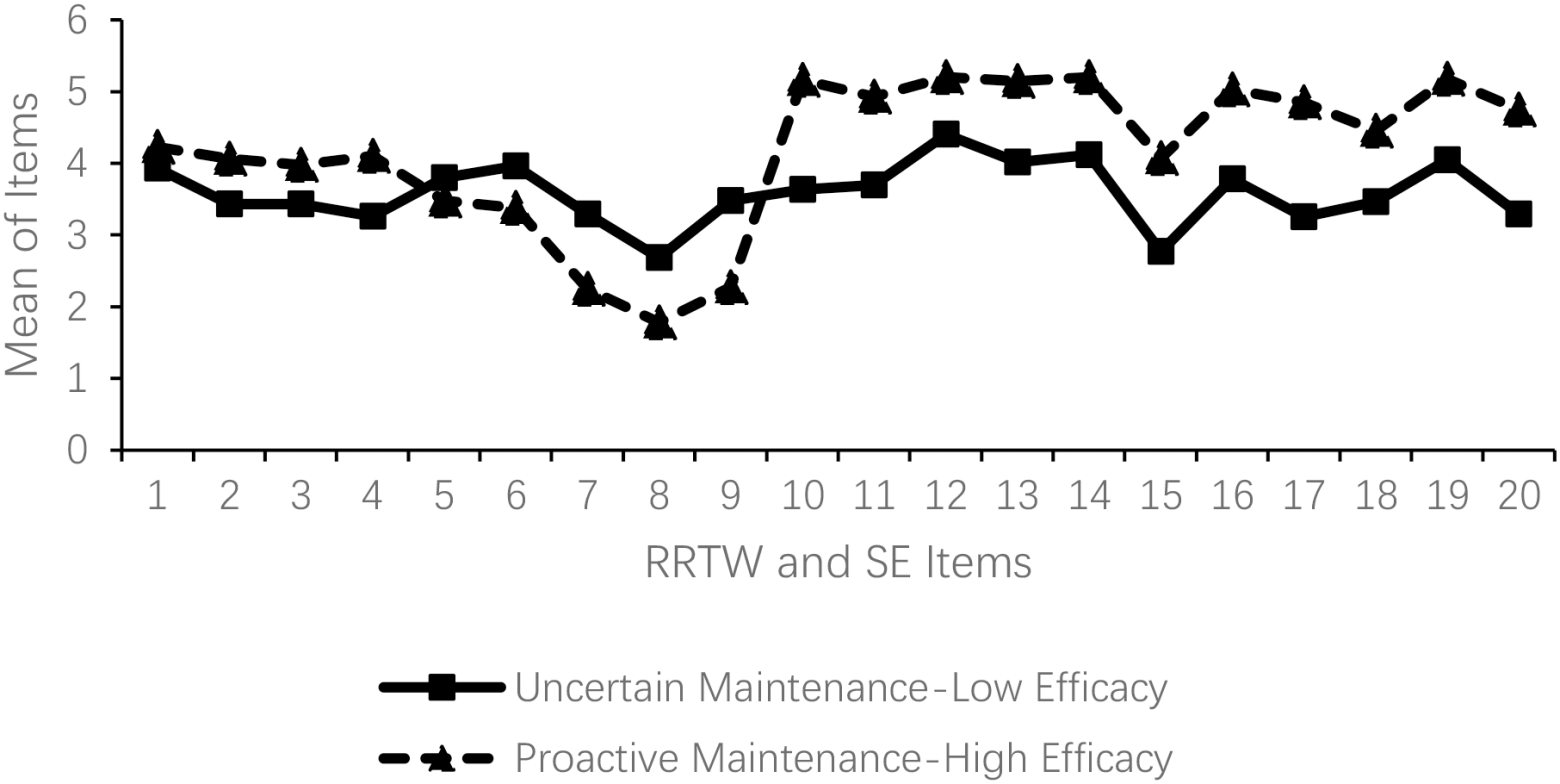
Mean item scores of the two latent profiles for the 20 items from the RRTW and RTW-SE scales.

The mean item scores for the two identified latent profiles among postoperative breast cancer patients who had returned to work are shown in **Figure 1**. Profile 1 was characterized by the highest scores on the Uncertain Maintenance dimension of readiness and comparatively low scores on self-efficacy. Therefore, it was labeled the “Uncertain Maintenance-Low Efficacy” group, comprising 34.68% of the total sample. In contrast, Profile 2 was characterized by the highest scores on the Proactive Maintenance dimension of readiness and high scores on self-efficacy. Consequently, it was named the “Proactive Maintenance-High Efficacy” group, accounting for 65.32% of the participants. **Table 3** presents the mean and standard deviation of each of the 20 indicators for the two latent classes, together with the scale/dimension to which each indicator belongs (Proactive Maintenance, Uncertain Maintenance, or RTW−SE). As expected, Class 2 (Proactive Maintenance−High Efficacy) showed higher means on Proactive Maintenance and self−efficacy items, whereas Class 1 (Uncertain Maintenance−Low Efficacy) showed higher means on Uncertain Maintenance items, confirming the validity of the class labels.

**Table 3:** Means (± SD) of each indicator by latent class and corresponding dimension.

Items	Scale/dimension	Uncertain maintenance-low efficacy (n=60) mean ± SD	Proactive maintenance-high efficacy (n=113) mean ± SD
Proactive maintenance (RRTW)			
item 1	RRTW – Proactive	3.93 ± 0.54	4.23 ± 0.48
item 2	RRTW – Proactive	3.43 ± 0.75	4.07 ± 0.72
item 3	RRTW – Proactive	3.43 ± 0.76	3.98 ± 0.83
item 4	RRTW – Proactive	3.26 ± 0.81	4.10 ± 0.65
Uncertain maintenance (RRTW)			
item 5	RRTW – Uncertain	3.80 ± 0.83	3.48 ± 0.98
item 6	RRTW – Uncertain	3.96 ± 0.86	3.37 ± 1.06
item 7	RRTW – Uncertain	3.29 ± 0.98	2.25 ± 0.96
item 8(reverse)	RRTW – Uncertain	2.68 ± 0.86	1.78 ± 0.61
item 9	RRTW – Uncertain	3.48 ± 1.04	2.27 ± 0.91
Return-to-work self-efficacy (RTW-SE)			
item10	RTW-SE	3.64 ± 1.20	5.16 ± 0.58
Item11(reverse)	RTW-SE	3.70 ± 1.12	4.92 ± 1.00
Item12	RTW-SE	4.40 ± 0.88	5.20 ± 0.57
Item13	RTW-SE	4.01 ± 1.03	5.15 ± 0.67
Item14	RTW-SE	4.12 ± 0.87	5.20 ± 0.61
Item15(reverse)	RTW-SE	2.77 ± 1.17	4.07 ± 1.43
Item16	RTW-SE	3.78 ± 1.02	5.03 ± 0.77
Item17	RTW-SE	3.25 ± 1.21	4.85 ± 0.93
Item18(reverse)	RTW-SE	3.46 ± 1.28	4.45 ± 1.23
item19	RTW-SE	4.05 ± 0.69	5.17 ± 0.52
Item20	RTW-SE	3.29 ± 1.27	4.75± 0.97

### 3.4 Correlation analysis among proactive maintenance, uncertain maintenance, and self-efficacy

The correlation analysis revealed a significant positive correlation between proactive maintenance and self-efficacy (*r* = 0.593, *p* < 0.001). Conversely, a significant negative correlation was found between uncertain maintenance and self-efficacy (*r* = -0.692, *p* < 0.001). This indicates that individuals with higher levels of uncertain maintenance tend to have lower self-efficacy. Detailed results are presented in **Table 4**.

**Table 4:** Correlation analysis among proactive maintenance, uncertain maintenance, and self-efficacy.

		Proactive maintenance	Uncertain maintenance	RTW-SE
Proactive maintenance	Pearson Correlation	1	-.325**	.593**
	Sig. (2-tailed)		<0.001	<0.001
	N	173	173	173
Uncertain maintenance	Pearson Correlation	-.325**	1	-.692**
	Sig. (2-tailed)	<0.001		<0.001
	N	173	173	173
RTW-SE	Pearson Correlation	.593**	-.692**	1
	Sig. (2-tailed)	<0.001	<0.001	
	N	173	173	173

**. Correlation is significant at the 0.01 level (2-tailed).

### 3.5 Univariate analysis of factors influencing latent profiles of RRTW and RTW-SE

Statistically significant differences were found in age, daily working hours, employment status and job satisfaction between the two latent profiles of postoperative breast cancer patients (all P < 0.05), as detailed in **Table 5**.

**Table 5:** Univariate analysis of factors influencing the latent profiles of RRTW and RTW-SE in patients who have returned to work.

Variable		Total (N)	Uncertain maintenance-low efficacy(N = 60)	Proactive maintenance-high efficacy(N = 113)	Statistic	*P*
Age (mean ± SD)		41.14 ± 6.16	39.68 ± 6.56	41.91 ± 5.83	-2.291	0.023*
Education	*-Elementary school or below*	2	2(3.33)	0(0.00)	9.271	0.099
	*- Junior high school*	13	2(3.33)	11(9.73)		
	*-High school/vocational*	11	2(3.33)	9(7.96)		
	*- College*	38	11(18.33)	27(23.89)		
	*- Bachelor’s degree*	94	36(60.00)	58(51.33)		
	*- Master’s degree or above*	15	7(11.67)	8(7.08)		
Ethnicity	*-Han*	167	58(96.67)	109(96.46)	2.948	0.567
	*-Tibetan*	1	1(1.67)	0(0.00)		
	*-Yi*	1	0(0.00)	1(0.88)		
	*-Hui*	1	0(0.00)	1(0.88)		
	*-others*	3	1(1.67)	2(1.77)		
Marital Status	*- Single*	11	7(11.67)	4(3.54)	4.371	0.112
	*- Married*	149	49(81.67)	100(88.50)		
	*- Divorced*	13	4(6.67)	9(7.96)		
Per Capita Household Income	*≤3000*	6	2(3.33)	4(3.54)	6.585	0.160
	*3000-5000*	26	8(13.33)	18(15.93)		
	*5000-8000*	35	17(28.33)	18(15.93)		
	*8000-10000*	27	12(20.00)	15(13.27)		
	*≥10000*	79	21(35.00)	58(51.33)		
Personality	*-Typical Introvert*	8	3(5.00)	5(4.42)	9.188	0.057
	*-Introvert Leaning*	33	17(28.33)	16(14.16)		
	*-Intermediate*	83	27(45.00)	56(49.56)		
	*-Extrovert Leaning*	40	13(21.67)	27(23.89)		
	*-Typical Extrovert*	9	0(0.00)	9(7.96)		
Work Type	*- Physical labor*	6	0(0.00)	6(5.31)	3.332	0.189
	*- Mental labor*	110	39(65.00)	71(62.83)		
	*- Mixed labor*	57	21(35.00)	36(31.86)		
Employment Contract	*- Permanent staff*	46	16(26.67)	30(26.55)	4.469	0.107
	*-Contract-based staff*	99	39(65.00)	60(53.10)		
	*- Other employment types*	28	5(8.33)	23(20.35)		
Years of employment, yr	*/*	17.24 ± 8.12	16.06 ± 7.55	17.87 ± 8.37	-1.396	0.165
Professional Title	*- None*	84	23(38.33)	61(53.98)	6.959	0.138
	*- Junior Title*	18	10(16.67)	8(7.08)		
	*- Intermediate Title*	46	17(28.33)	29(25.66)		
	*- Senior Title*	20	9(15.00)	11(9.73)		
	*- Other*	*5*	*1(1.67)*	*4(3.54)*		
Employment status	*-Partial return to work*	89	42(70.00)	47(41.59)	13.907	0.001*
	*-Full return to work*	73	14(23.33)	59(52.21)		
	*-New job*	11	4(6.67)	7(6.19)		
Work Hours	*- <6 h/day*	*18*	*5(8.33)*	*13(11.50)*	9.044	0.011*
	*- 6–8 h/day*	97	26(43.33)	*71(62.83)*		
	*- >8 h/day*	58	29(48.33)	29(25.66)		
Workload	*- Low*	10	1(1.67)	9(7.96)	3.590	0.166
	*- Moderate*	109	37(61.67)	72(63.72)		
	*- High*	54	22(36.67)	32(28.32)		
Job Satisfaction	*- Very dissatisfied*	6	3(5.00)	3(2.65)	20.216	<0.001*
	*- Dissatisfied*	12	8(13.33)	4(3.54)		
	*- Neutral*	56	28(46.67)	28(24.78)		
	*- Satisfied*	90	20(33.33)	70(61.95)		
	*- Very satisfied*	9	1(1.67)	8(7.08)		
Disease Duration	*- <1 year*	92	31(51.67)	61(53.98)	0.817	0.665
	*- 1–3 years*	68	23(38.33)	45(39.82)		
	*- >3 years*	13	6(10.00)	7(6.19)		
TNM Stage	*- Stage I*	47	16(26.67)	31(27.68)	0.812	0.847
	*- Stage II*	86	29(48.33)	57(50.89)		
	*- Stage III*	21	7(11.67)	14(12.50)		
	*- Unknown*	18	8(13.33)	10(8.93)		
Radiotherapy	*No*	47	16(26.67)	31(27.43)	0.012	0.914
	*Yes*	126	44(73.33)	82(72.57)		
Chemotherapy	*No*	26	8(13.33)	18(15.93)	0.207	0.649
	*Yes*	147	52(86.67)	95(84.07)		
Endocrine therapy	*No*	55	15(25.00)	40(35.40)	1.954	0.162
	*Yes*	118	45(75.00)	73(64.60)		
Immunotherapy/Targeted therapy	*No*	125	44(73.33)	81(71.68)	0.053	0.817
	*Yes*	48	16(26.67)	32(28.32)		
Surgery Type	*- Breast-conserving*	64	23(38.33)	41(36.28)	0.071	0.965
	*- Mastectomy*	44	15(25.00)	29(25.66)		
	*- Reconstruction*	65	22(36.67)	43(38.05)		
Lymph Dissection	*- None*	12	4(6.67)	8(7.08)	0.213	0.899
	*- Sentinel*	91	33(55.00)	58(51.33)		
	*- Dissection*	70	23(38.33)	47(41.59)		

An asterisk (*) indicates statistical significance at p < 0.05*; Tests used: chi-square test for categorical variables; independent-samples t-test for continuous variables; No missing data were present in any of the variables used in the analysis. All variables had complete records;.

### 3.6 Multivariate analysis of factors influencing the latent profiles of RRTW and RTW-SE

To identify factors associated with latent profile membership, a binary logistic regression model was performed. The outcome variable was defined based on the latent profile analysis (LPA) results: participants classified into the “Proactive Maintenance−High Efficacy” profile were coded as 1, and those in the “Uncertain Maintenance−Low Efficacy” profile as 0. Candidate independent variables were selected based on univariate comparisons (p < 0.05), which included age(continuous), daily working hours (<6h, 6-8h, reference>8h), employment status (partial/full/new job, reference new job), and job satisfaction (ordinal 1–5). The coding schemes for these variables are presented in **Table 6**.

**Table 6:** Coding schemes for the independent variables in the analysis of factors associated with RRTW and RTW-SE.

Independent variable	Assignment method
Work hours	Work Hours Dummy-coded with “>8h” as the reference: <6h = (1,0,0); 6~8h = (0,1,0); >8h = (0,0,0)
Employment status	Employment status Dummy-coded with “new job” as the reference: Partial return to work = (1,0,0); Full return to work = (0,1,0); new job = (0,0,0)
Age	Continuous, reported in years
Job satisfaction	Ordinal variable, coded as 1–5 (1 = Very Dissatisfied, 2 = Dissatisfied, 3 = Neutral, 4 = Satisfied, 5 = Very Satisfied)

Dummy coding was applied for categorical variables, with the reference category coded as 0 across all comparisons.

The logistic regression analysis identified daily working hours, age and job satisfaction as significant independent factors influencing the latent profiles among postoperative breast cancer patients. The Hosmer-Leme show goodness-of-fit test yielded a χ² value of 7.366 (P = 0.498), indicating that the model demonstrated a good overall fit. Detailed results of the multivariate analysis are provided in **Table 7**.

**Table 7:** Multivariate analysis of factors influencing the latent profiles of RRTW and RTW-SE.

Independent variable	β value	SE value	Wald χ² value	P-value	OR(95%CI)
Work hours (<6hours per day)	1.153	0.684	2.844	0.092	3.167(0.830, 12.094)
Work hours (6–8 hours per day)	0.834	0.397	4.415	0.036*	2.303(1.058, 5.015)
Job satisfaction	0.731	0.232	9.966	0.002*	2.078(1.319, 3.271)
Age	0.060	0.029	4.208	0.040*	1.062(1.003, 1.125)
Employment status (Partial return to work)	-0.618	0.706	0.766	0.381	0.539(0.135, 2.150)
Employment status (Full return to work)	0.953	0.737	1.672	0.196	2.593(0.612, 10.989)
Constant	-4.891	1.633	8.972	0.003*	0.008

*Statistically significant, p < 0.05 (logistic regression); SE, Standard error.

## 4 Discussion

### 4.1 Individual heterogeneity in RRTW and RTW-SE among postoperative breast cancer patients who have returned to work

This study identified two distinct latent profiles of RRTW and RTW-SE among postoperative breast cancer patients who have returned to work—namely, the “Uncertain Maintenance-Low Efficacy” group and the “Proactive Maintenance-High Efficacy” group—indicating the presence of significant individual heterogeneity. Furthermore, a positive correlation was observed between the Proactive Maintenance dimension and self-efficacy (*r* = 0.593), suggesting that individuals with higher levels of proactive maintenance tend to possess greater self-efficacy (i.e., confidence in their own abilities). Conversely, a strong negative correlation was found between the Uncertain Maintenance dimension and self-efficacy (*r* = -0.692), indicating a close and mutually inhibitory relationship: high levels of uncertain maintenance may accompany reduced self-efficacy, while low self-efficacy may, in turn, be associated with worsened patterns of uncertain coping. These findings underscore that self-efficacy is positively associated with sustaining employment after returning to work.

This result is consistent with the study by Han Fang et al. (27), which reported a close correlation between RTW-SE and patients’ RRTW. Patients with high self-efficacy often exhibit greater confidence about returning to their jobs, which is associated with taking more active steps and higher levels of readiness. Self-efficacy also plays a crucial role in alleviating anxiety and fear, maintaining motivation, promoting work participation, and improving work efficiency (28).

Meanwhile, the “Proactive Maintenance-High Efficacy” profile accounted for 65.32% of the sample, indicating that the majority of postoperative breast cancer patients who returned to work were in a stage of proactive maintenance regarding their RRTW and exhibited a high level of RTW-SE. A defining characteristic of this stage is that most patients have found a balance between work and their illness, which allows them to effectively manage their health conditions while maintaining or engaging in work activities. This also demonstrates that, despite the numerous challenges faced by cancer survivors, many can achieve a work-life balance through proactive efforts and self-adjustment (29).

Conversely, 34.68% of patients belonged to the “Uncertain Maintenance-Low Efficacy” profile, with a mean RRTW score of 17.21 ± 1.50. This proportion is consistent with the findings of Wang Renxiu (30), whose study suggested that even after returning to work, patients often experience uncertainty about their cancer prognosis and worry about potential future absences, which is associated with poorer concentration at work and may be negatively associated with their overall job performance and quality of life. Similarly, Dai Jianjuan et al. (31) found that return-to-work readiness is a significant factor influencing the work-related quality of life in lung cancer patients after they resume work.

In addition to identifying latent profile characteristics, this study further identified key factors associated with RRTW and RTW-SE among postoperative breast cancer patients who have returned to work through logistic regression analysis.

### 4.2 Factors associated with RRTW and RTW-SE in postoperative breast cancer patients

#### 4.2.1 Daily working hours

The logistic regression model in this study identified daily working hours as the most significant factor associated with RRTW and RTW-SE. Specifically, patients working more than 8 hours per day were associated with significantly lower readiness and self-efficacy after returning to work compared to those working 6–8 hours per day. This finding is consistent with the study by Zhang Yue (32), which reported that a weekly workload exceeding 40 hours was a risk factor for the return-to-work readiness of young and middle-aged patients with rhegmatogenous retinal detachment (RRD). Furthermore, Yang et al. (4) also indicated that the work capacity of postoperative breast cancer patients declines to varying degrees due to disease treatment and its long-term effects, rendering them unable to sustain long-hour or high-intensity physical labor. Studies have shown that most breast cancer patients experience some level of fear of cancer recurrence post-surgery (33). On this context, the concern over potential recurrence, when combined with prolonged working hours, may be associated with higher psychological stress and poorer mental state, which in turn are related to lower self-efficacy.

#### 4.2.2 Age

The regression model in this study showed that older patients were associated with higher levels of RRTW and RTW-SE. This finding aligns with the results reported by Fan Rongrong (34) and Ahn et al. (35, 36), which demonstrated a positive correlation between age and return to work, but contrasts with the findings of Yang Lijun et al. (37, 38). Chen et al. (39) found that younger breast cancer patients often exhibit extreme despair and fear towards the disease, generally lacking confidence in returning to work. Furthermore, studies by Tang Jie et al. (40–42) also indicated that younger patients experience stronger feelings of stigma, often leading to lower psychosocial adaptation. Their level of work engagement after returning to work is also relatively low. A possible explanation is that most younger breast cancer patients are at a critical stage of career development and family establishment, with higher aspirations and pursuits for both work and life. Increased workload and competitive pressure raise concerns about their ability to cope effectively. Coupled with image disturbance and functional limitations caused by cancer, these factors can foster fear and anxiety about returning to work, thereby predisposing them to work withdrawal behavior (43). In contrast, older patients, with richer life experiences, tend to possess more mature and composed psychological coping abilities and strategies. They are better equipped to make rational decisions when facing pressure, consequently enhancing their RRTW and RTW-SE (44).

#### 4.2.3 Job satisfaction

The logistic regression model revealed that patients who were highly satisfied with their pre-diagnosis jobs were associated with greater readiness and self-efficacy upon returning to work compared to those who were dissatisfied. Job satisfaction reflects an individual’s attitude toward their work; high satisfaction indicates a positive attitude and emotional attachment, whereas dissatisfaction suggests passive engagement and presenteeism. Coupled with physical discomfort, this lack of satisfaction is associated with lower enthusiasm and motivation, which in turn are related to reduced readiness and self-efficacy for work resumption. This finding is consistent with Chang Lixia (45), who reported that breast cancer patients with moderate job satisfaction scored lower on work engagement scales than those reporting high satisfaction. Furthermore, studies have confirmed that job satisfaction is a significant predictor of whether patients will return to work (46). Breast cancer survivors who successfully resume employment typically report higher job satisfaction and maintain a positive, optimistic, and competitive attitude toward their work (47), which is associated with higher readiness and self-efficacy during the RTW process.

Significant heterogeneity persists in RRTW and RTW-SE among breast cancer patients who have returned to work post-surgery. Healthcare professionals should prioritize and actively address these patients’ readiness and self-efficacy levels by identifying their specific profiles and providing tailored, individualized occupational education and support.

For patients with high self-efficacy in the “Proactive Maintenance” phase, healthcare providers should monitor disease progression and psychological changes through postoperative follow-ups, assisting them in sustaining higher levels of work readiness and enabling them to confidently navigate work and life challenges.

For patients with low self-efficacy in the “Uncertain Maintenance” phase, interventions should focus on enhancing self-efficacy to rebuild confidence and reduce stigma. Additionally, professional prognostic guidance should be provided promptly to address disease-related concerns in the workplace, fostering accurate illness perceptions and mitigating uncertainties about maintaining employment.

Furthermore, healthcare professionals should provide targeted psychological support based on the patient’s age. For younger patients, they should be encouraged to actively face work-related challenges, provided with emotional support, and guided to adopt optimistic and positive attitudes toward both their disease and employment. Younger breast cancer survivors should be supported in their RTW. For older patients, although they generally demonstrate higher levels of RRTW and RTW-SE, it remains crucial to monitor their physical and psychological load after resuming work. Elevated levels of mental workload and physical demands can still adversely affect their health (44). Necessary support should be provided when required.

Concurrently, tailored rehabilitation and work adaptation plans should be developed, particularly for patients working more than 8 hours daily. For those engaged in physically demanding jobs, focus should be placed on their physical recovery, providing appropriate exercise recommendations. For individuals in mentally demanding occupations, attention should be directed toward their psychological rehabilitation and cognitive recovery. Patients should be encouraged to negotiate with their employers regarding working hours and potential job repositioning to more suitable roles. Besides, relevant departments should also prioritize this population of returning breast cancer survivors by refining work policies and healthcare security systems. This will help patients feel valued by society, thereby enhancing their job satisfaction (46).

Furthermore, studies have shown that social support can ensure good maintenance of post-disease return−to−work (RTW) status (18). In view of this, out−of−hospital transitional care should be actively implemented for patients (48), with dynamic assessment of their rehabilitation progress, timely provision of rehabilitation advice tailored to the working phase, psychological support, and continuous improvement of rehabilitation strategies. These measures may contribute to enhancing the long−term outcomes of occupational function maintenance (18). Specifically, establishing a postoperative rehabilitation guidance and consultation platform for breast cancer survivors, along with offering educational lectures on disease−related knowledge, may effectively improve patients’ rehabilitation capacity and their understanding of the disease. In turn, this may alleviate fear and anxiety regarding disease progression and facilitate sustained work maintenance (18).

## 5 Limitations

This study has several limitations. First, the sample was recruited from a single tertiary hospital using convenience sampling and the sample size is relatively modest, which may limit the generalizability of the findings. Our sample was predominantly highly educated (54% held a bachelor’s degree or higher) and engaged in mental labor (63.58%), which may not represent breast cancer patients with lower educational levels or those in physically demanding occupations. Second, patients who were unable to complete the questionnaires independently received assistance from the investigators. Although a standardized script was used, this may have introduced response bias, as patients’ answers could have been influenced by the investigator’s presence or wording. Third, the cross-sectional design precludes any causal inferences; only associative relationships can be drawn. Therefore, our findings—particularly the identified latent profiles and associated factors—should be considered preliminary and require validation in more diverse, multi-center populations with larger sample sizes. Future research should adopt prospective, multi-center designs and include patients with varied socioeconomic and occupational backgrounds to improve generalizability. Furthermore, subsequent studies could consider integrating quantitative and qualitative approaches to gain a more comprehensive understanding of the current status and determinants of RRTW and RTW-SE in this population, which may inform more targeted clinical interventions.

## 6 Conclusion

This study demonstrates significant population heterogeneity in RRTW and RTW-SE among postoperative breast cancer patients who have returned to work, identifying two distinct profiles: “Proactive Maintenance-High Efficacy” and “Uncertain Maintenance-Low Efficacy.” These profiles were significantly associated with daily working hours, age, and job satisfaction. The findings suggest that clinical practice and occupational support may benefit from a more individualized approach. Particular attention should be given to younger patients, those with longer working hours, and those with lower job satisfaction. Tailored interventions focusing on optimizing work systems, enhancing work-related confidence, and improving job satisfaction may be important to promote sustainable work reintegration. Future research should employ prospective designs to further validate the effectiveness of such targeted interventions.

## Data Availability

The raw data supporting the conclusions of this article will be made available by the authors, without undue reservation.

## References

[1] Lei S Zheng R Zhang S Chen R Wang S Sun K . Breast cancer incidence and mortality in women in China: temporal trends and projections to 2030. Cancer Biol Med. (2021) 18:900–9. doi: 10.20892/j.issn.2095-3941.2020.0523 34002584 PMC8330522

[2] Kiasuwa Mbengi RL Nicolaie AM Goetghebeur E Otter R Mortelmans K Missinnne S . Assessing factors associated with long-term work disability after cancer in Belgium: a population-based cohort study using competing risks analysis with a 7-year follow-up. BMJ Open. (2018) 8:e014094. doi: 10.1136/bmjopen-2016-014094 29455161 PMC5855469

[3] Butow P Laidsaar-Powell R Konings S Lai CH Ho CL Wang CC . Return to work after a cancer diagnosis: a meta-review of reviews and a meta-synthesis of recent qualitative studies. J Cancer Surviv. (2020) 14:114–34. doi: 10.1007/s11764-019-00828-z 31858379

[4] Yang ZY Chen WL Wu WT Lai CH Ho CL Wang CC . Return to work and mortality in breast cancer survivors: a 11-year longitudinal study. Int J Environ Res Public Health. (2022) 19:14418. doi: 10.3390/ijerph192114418 36361291 PMC9655987

[5] Cao HL . The Chinese localization of the readiness for return-to-work scale and the application in breast cancer patients. Zhengzhou: Zhengzhou University (2018).

[6] Chen SN Hou YT Li Z Yao QQ Liu ST Gao M . Work-related quality of life and its influencing factors in breast cancer patients after returning to work post-surgery. Chin Ment Health J. (2024) 38:493–9.

[7] Petersen KS Madsen LS Nielsen CV Labriola M Stapelfeldt CM . To have and then lose the safety net"-female cancer survivors' experiences of the process of becoming ready to return to work. Work. (2021) 70:1121–30. doi: 10.3233/WOR-213623 34864711

[8] Wang LY . Analysis of the status and influencing factors of readiness for return-to-work in young and middle-aged colorectal cancer patients after surgery. Yan'an: Yan'an University (2021).

[9] Franche RL Krause N . Readiness for return-to-work following injury or illness: conceptualizing the interpersonal impact of health care, workplace, and insurance factors. J Occup Rehabil. (2002) 12:233. doi: 10.1023/a:1020270407044 12389476

[10] Brathen TN Brage S Tellnes C Fugelli P . A prospective study of the association between the readiness for return to work seale and future work participation in Norway. J Occup Rehabil. (2014) 24:650–7. doi: 10.1007/s10926-013-9497-y 24395042

[11] Wolvers M Leenseen M Groeneveld IF Frings-Dresen MH De Boer AG . Predictors for earlier return to work of cancer patients. J Cancer Survive. (2018) 12:169–77. doi: 10.1007/s11764-017-0655-7 29076003 PMC5884890

[12] Rosbjerg R Sung H Zachariae R Mikkelsen EM Homoe P Larsen MB . The predictive value of return-to-work self-efficacy for return to work among employees with cancer undergoing chemotherapy. J Occup Rehabil. (2020) 30:665–78. doi: 10.1007/s10926-020-09882-2 32114672 PMC7716905

[13] Hartke RJ Trierweiler R Bode R . Critical factors related to return to work after stroke: a qualitative study. Top Stroke Rehabil. (2011) 18:341–51. doi: 10.1310/tsr1804-341 21914598

[14] Wang YX . Analysis of influencing factors on the readiness of young and middle-aged patients to return to work after PCI. Guangzhou: Guangzhou University of Chinese Medicine (2024). doi: 10.27044/d.cnki.ggzzu.2024.000909

[15] Cheng H . Correlation study on social support, sense of coherence, and readiness for return-to-work in breast cancer patients. Suzhou: Soochow University (2023). doi: 10.27351/d.cnki.gszhu.2023.001348

[16] Pan SW . Analysis of the quo and influencing factors of the Readiness for Return-To-Work of young and middle-aged patients with pulmonary tuberculosis. Nanjing: Nanjing University of Chinese Medicine (2025). doi: 10.27253/d.cnki.gnjzu.2025.000439

[17] Liu F Zhang Z Lin B . Assessing the psychometric properties of the Chinese Return-to-Work Self-Efficacy Questionnaire using Rasch model analysis. Health Qual Life Outcomes. (2022) 20:27. doi: 10.1186/s12955-022-01929-7 35172850 PMC8848945

[18] Wang SS . Construction of prediction models for readiness and maintenance to return to work in young and middle-aged. Guangzhou: Guangzhou Medical University (2025). doi: 10.27043/d.cnki.ggzyc.2025.000482

[19] Black O Keegel T Sim MR Collie A Smith P . The effect of self-efficacy on return-to-work outcomes for workers with psychological or upper-body musculoskeletal injuries: a review of the literature. Occup Rehabil. (2018) 28:16–2. doi: 10.1007/s10926-017-9697-y 28271400

[20] Hu YY Mao FY Zhang J Yu L Wu Q . Influencing factors of return to work among young and middle-aged patients with coronary heart disease. J Nurs Sci. (2022) 37. doi: 10.3389/fmed.2026.1795966 42210934 PMC13212131

[21] Wang SQ . A latent profile analysis and study of influencing factors on nurse presenteeism based on the Effort-Reward Imbalance model. Shenyang: China Medical University (2024).

[22] Wang MC Bi XY . Latent variable modeling and mplus applications—advanced edition. Chongqing: Chongqing University Press (2018). p. 13–5.

[23] Buckner TW Wang J Dewalt DA Jacobs S Reeve BB . Patterns of symptoms and functional impairments in children with cancer. Pediatr Blood Cancer. (2014) 61:1282–8. doi: 10.1002/pbc.25029 24634396 PMC4412461

[24] Ni P Chen JL Liu N . Sample size estimation in quantitative nursing research. Chin J Nurs. (2010) 45:378–80.

[25] Franche RL Corbiere M Lee H Breslin FC Hepburn CG . The Readiness for Return-To-Work (RRTW) scale: development and validation of a self-report staging scale in lost-time claimants with musculoskeletal disorders. J Occup Rehabil. (2007) 17:450–72. doi: 10.1007/s10926-007-9097-9 17701326

[26] Lagerveld SE Bultmann U Franche RL Van Dijk FJH Vlasveld MC van der Feltz-Cornelis CM . Factors associated with work participation and work functioning in depressed workers: a systematic review. J Occup Rehabil. (2010) 20:275–92. doi: 10.1007/s10926-009-9224-x 20091105 PMC2923705

[27] Han F Guo YJ Dai Q Dai Q Xue Q Xu JS . Status quo of readiness for return-to-work and its influencing factors in cancer patients during rehabilitation. J Nurs. (2021) 38:34–7.

[28] Ying X Wei QF Li DF Cheng Y . Relationship between fear of cancer progression, death anxiety, and cancer self-efficacy in lung cancer survivors. J Nurs Manage. (2022) 22:392–7.

[29] Yin YY Long XX Zhang J Yang M . Status and influencing factors of work readiness in postoperative lung cancer patients who have returned to work. Chin J Clinical Thoracic Cardiovascular Surg. (2025). p. 1–7.

[30] Wang RX . Study on the status and influencing factors of readiness for return-to-work in patients after coronary stent implantation. Jinan: Shandong University of Traditional Chinese Medicine (2021).

[31] Dai JJ Wang W Yan BE Wu DD Yuan XY Chen ZJ . Status and influencing factors of work-related quality of life in lung cancer patients returning to work. Chin J Gen Pract. (2024) 22:513–6.

[32] Zhang Y . A study on the correlation between social support, illness perception, hope, and readiness for return-to-work in young and middle-aged patients with rhegmatogenous retinal detachment. Tianjin: Tianjin University of Traditional Chinese Medicine (2022).

[33] He YF Sun T Hu LY Ji X Zhao XJ . Relationship between social and psychological factors and fear of cancer recurrence in breast cancer patients. J Int Psychiatry. (2024) 51:1877–9.

[34] Fan RR Li XY Li JH Luo L Tan Y . Study on the status and influencing factors of readiness for return-to-work in breast cancer survivors. Evidence-Based Nurs. (2021) 7:2198–204.

[35] Ahn E Cho J Shin DW Park BW Ahn SH Noh DY . Impact of breast cancer diagnosis and treatment on work-related life and factors affecting them. Breast Cancer Res Treat. (2009) 116:609–16. doi: 10.1007/s10549-008-0209-9 18855135

[36] Schmidt ME Scherer S Wiskemann J Steindorf K . Return to work after breast cancer: the role of treatment-related side effects and potential impact on quality of life. Eur J Cancer Care. (2019) 28:e13051. doi: 10.1111/ecc.13051 31033073

[37] Yang LJ Lin ZZ Wang YB Wang WQ Ye HZ . Relationship between readiness for return-to-work and family resilience in breast cancer patients. Chin J Modern Nurs. (2020) 26:2055–60.

[38] Yang SS Liu JE Su YL Zhao YQ Cui J Wang L . Analysis of return-to-work status and influencing factors in breast cancer patients. Chin Nurs Manage. (2020) 20:821–5.

[39] Chen HL Zhou MQ Tian W Meng KX He HF . Effect of age on breast cancer patient prognoses: a population-based study using the SEER 18 database. PloS One. (2016) 11:e0165409. doi: 10.1371/journal.pone.0165409 27798652 PMC5087840

[40] Tang J Wang DH . Research progress on stigma in female breast cancer patients. J Nurs Manage. (2018) 18:576–9.

[41] Yin CL Qu HL Yang FG Ren HQ Leng HP . Study on the status and influencing factors of psychosocial adaptation in young breast cancer patients after surgery. J Nurs Manage. (2019) 19:457–61.

[42] Zhang YD Liu RH Hu YJ . Study on the status of self-transcendence and its impact on work engagement in young and middle-aged breast cancer survivors. J Nurs. (2021) 38:27–30.

[43] Ma Y Zhang MY Wang T Bi HM . Research progress on stigma in breast cancer patients. Gen Nurs. (2019) 17:4021–3.

[44] Guo PL Ge GQ Ma XX . Study on work-related mental load and its influencing factors in breast cancer patients returning to work. Occupation Health. (2020) 36:2065–9.

[45] Chang LX . Analysis of work engagement status and influencing factors in young and middle-aged breast cancer patients returning to work. Jinan: Shandong University (2023).

[46] Zhang Y Cao SQ Zhang SQ Zhu YQ Li MY Yan DX . Research progress on influencing factors and interventions for readiness for return-to-work in young and middle-aged patients. Occupation Health. (2022) 38:2431–9.

[47] Heinesen E Kolodziejczyk C Ladenburg J Andersen I Thielen K . Return to work after cancer and pre-cancer job dissatisfaction. Appl Econ. (2017) 49:4982–98. doi: 10.1080/00036846.2017.1296555 37339054

[48] Zhao Q Wu Q Sun X Niu SZ Shu JJ Mao YF . Development of evaluation index system of continuous care for stroke patients. Chin J Nurs. (2020) 55:171–6.

